# Label-free, simultaneous quantification of starch, protein and triacylglycerol in single microalgal cells

**DOI:** 10.1186/s13068-017-0967-x

**Published:** 2017-11-17

**Authors:** Yuehui He, Peng Zhang, Shi Huang, Tingting Wang, Yuetong Ji, Jian Xu

**Affiliations:** 1 Single-Cell Center, CAS Key Laboratory of Biofuels and Shandong Key Laboratory of Energy Genetics, Qingdao Institute of BioEnergy and Bioprocess Technology, Chinese Academy of Sciences, Qingdao, Shandong China; 2 University of Chinese Academy of Sciences, Beijing, China; 3 Laboratory for Marine Biology and Biotechnology, Qingdao National Laboratory for Marine Science and Technology, Qingdao, Shandong China

**Keywords:** Single-cell Raman spectroscopy, Starch content, Protein content, Triacylglycerol content, Phenotypic heterogeneity, Sampling depth, *Chlamydomonas reinhardtii*, Microalgae

## Abstract

**Background:**

Current approaches for quantification of major energy-storage forms in microalgae, including starch, protein and lipids, generally require cell cultivation to collect biomass followed by tedious and time-consuming analytical procedures. Thus, label-free, non-destructive and simultaneous quantification of such macromolecules at single-cell resolution is highly desirable in microalgal feedstock development and bioprocess control.

**Results:**

Here, we established a method based on single-cell Raman spectra (SCRS) that simultaneously quantifies the contents of starch, protein, triacylglycerol (TAG) and lipid unsaturation degree in individual *Chlamydomonas reinhardtii* cells. Measurement accuracy for the contents based on full SCRS spectrum each reached 96.86–99.24%, all significantly higher than single peak-based models. However, accuracy and reliability of measurement are dependent on the number of cells sampled, thus a formal mathematical framework was proposed and validated to rationally define “minimal sampling depth” for a given state of cellular population. Furthermore, a barcode consisting of 13 marker Raman peaks was proposed to characterize the temporal dynamics of these energy-storage products, which revealed that the average contents of starch and TAG increased, while their heterogeneity indices decreased, with those of protein being exactly the opposite. Finally, our method is widely applicable, as measurements among cells from liquid suspension culture, wet paste and frozen dried powder all exhibited excellent consistency.

**Conclusions:**

When sampled at proper depth, SCRS can serve as a quantitative and generally applicable tool for characterization and screening of strains and bioprocesses based on the profile of energy-storage macromolecules and their among-cell heterogeneity.

**Electronic supplementary material:**

The online version of this article (10.1186/s13068-017-0967-x) contains supplementary material, which is available to authorized users.

## Background

The co-existence of multiple intracellular energy-storage forms and the interconversion among them are a common theme of microbial, plant or animal cells on Earth. For example, microalgae, a huge and diverse group of unicellular plants, can efficiently convert solar energy and carbon dioxide into a variety of co-present intracellular energy-dense macromolecules, which mainly include polysaccharides (e.g., starch), proteins and lipids (e.g., triacylglycerol, TAG) [1–3]. Thus, the ability to simultaneously measure the cellular contents of these compounds with high throughput and low cost is of value to strain development, process engineering and mechanistic studies of cell factories.

However, current approaches for profiling starch, protein and TAG contents in microalgae and plant cells generally consist of multiple tedious and time-consuming steps, including accumulation of biomass, extraction of metabolite mixtures from the bulk biomass and then quantification of the target compounds by separate assays. After the extraction of metabolite mixtures, the inherently inefficient “one procedure per target compound” paradigm was generally followed: (i) for starch, enzymatic conversion to glucose and then quantification by colorimetric analysis [4, 5]; (ii) for proteins, purification of proteins via alkaline lysis and then quantification by bicinchoninic acid-based spectrophotometric assays [6] and (iii) for TAG, conversion to fatty acid methyl esters (FAMEs) via transesterification and then quantification by gas chromatography–mass spectrometry (GC–MS) [7]. Notably, due to the requirement for significant amounts of microalgal biomass (typically at the µg–mg range) to start with, these approaches are dependent on a priori cultivation of cells. This limitation further slows down the analysis, hinders the increase of throughput and even renders the analysis impossible, as many or most of the microbial cells (including microalgae) in nature remain difficult to culture.

To tackle these challenges, single-cell-based methods for metabolite analysis have emerged [8, 9]. For example, oligosaccharides and flavonoids in single yeast cells were successfully analyzed by mass spectrometry (MS) [10]. However, MS-based methods usually require destructive and sophisticated sample preparation which excludes downstream additional analysis of genomes or transcriptomes. On the other hand, fluorescent protein-based sensors [11, 12] or aptamer-based technology [13, 14] were used to image proteins and nucleic acids such as calmodulin-binding peptides and ribozymes. However, these methods usually are applicable to only those cells that can be labeled and those metabolites that can be labeled quantitatively with fluorescence tags or aptamers. Clearly, single-cell technologies capable of simultaneously, rapidly and readily measuring starch, protein and TAG contents in a non-destructive, label-free and generally applicable manner are of great value.

Single-cell Raman spectra (SCRS) represent the collective Raman spectra of molecules in one cell and provide an intrinsic chemical profile of the cell in a label-free and non-destructive manner [15–17]. As each molecule carries characteristic Raman spectra, many studies have attempted to model the contents of certain molecules in an isogenic cell population based on a collection of SCRS randomly sampled from the population (also called a Ramanome; [18]). However, a number of key questions remain unanswered. (i) Most past studies have selected one or just a few Raman peaks (typically derived from Raman spectra of reference chemicals) for the quantification of target compounds, yet did not evaluate the reliability of such choices [19–21]. For example, the relative abundance of lipids, paramylon and chlorophyll in *Euglena gracilis* was estimated via 2850, 2910 and 2937 cm^−1^ [19], while that of lipids and astaxanthin in *Haematococcus pluvialis* was estimated via 1448 and 1520 cm^−1^ [20]; however, whether and to what degree these peaks can specifically quantify the target compounds were actually not assessed. (ii) Most studies that aimed for quantification only target one singular compound, such as the starch content in *Chlamydomonas reinhardtii* and *Chlorella pyrenoidosa* [22] or the TAG content in *Nannochloropsis oceanica* [23], yet it is not clear whether the cellular contents of the co-existent energy-storage compounds, e.g., starch, protein, TAG and others, can be simultaneously quantified. This is important as many factors including the potential overlaps of Raman bands assignment among compounds, choice of sample pre-treatment methods, parameters of Raman measurement and species-specific property of microalgae can all potentially limit the practicability and reliability of SCRS in generating the measurements in a quantitative and ‘landscape-like’ manner. (iii) To derive the overall content and its degree of variation for target molecules in a cellular population, most studies have either sampled cells at a very low sampling depth [24–26], i.e., the number of cells measured for SCRS (e.g., only three cells sampled from each population [24]), or have not provided any rationale for their choice of sampling depth [19, 22, 23, 27, 28]. In fact, the link between method performance and sample depth, an experimental parameter directly determining throughput and common to all SCRS-based experiments, has not been critically probed. (iv) Most studies have tested method performance on live single cells from suspended liquid cultures [21–24], and whether the method is robust under other frequently encountered storage conditions is not clear, which however can be a crucial limiting factor as living cells may be either unobtainable or of limited shelf live (thus, sample freezing might be inevitable before SCRS acquisition).

Here, by deep sampling the SCRS of *C. reinhardtii* at 16 time points over 8 days under nitrogen depletion, we established a method based on single-cell Raman spectra (SCRS) that simultaneously quantified the contents of starch, protein, triacylglycerol and lipid unsaturation degree in individual cells. The measurement accuracy for the contents based on full spectrum each reached 96.86–99.24%, all significantly higher than single peak-based models. However, accuracy and reliability of measurement are dependent on the number of cells sampled, thus a formal mathematical framework was proposed and validated to rationally define “minimal sampling depth” for a given state of cellular population. Furthermore, a barcode consisting of 13 marker Raman peaks was proposed to characterize the temporal dynamics of these energy-storage products, where the average contents of starch and TAG increased while their heterogeneity indices decreased, with those of protein being exactly the opposite. Finally, measurements among cells from liquid suspension culture, wet paste and frozen dried powder exhibited excellent consistency, suggesting applicability under a wide range of cell-storage conditions. Thus SCRS, when sampled at proper depth, can serve as a quantitative and generally applicable tool for the characterization and screening of strains and bioprocesses based on cellular biosynthetic profile and its among-cell heterogeneity.

## Results

### Dynamics of starch, protein and TAG contents and lipid unsaturation degree at the population level as measured by conventional approaches

**Fig. 1:**
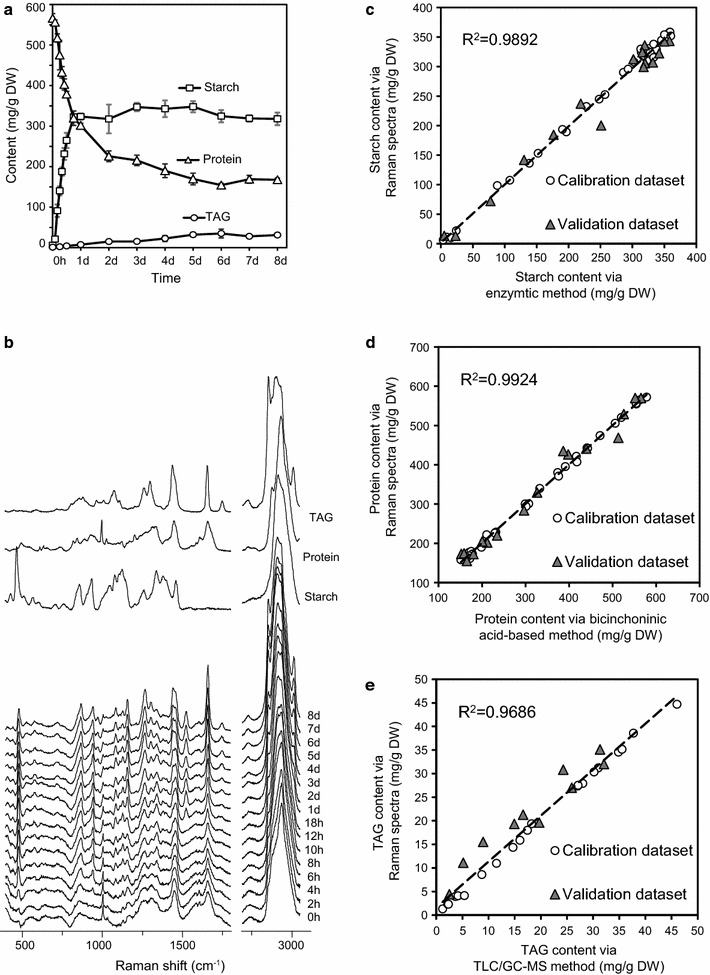
Quantification of starch, protein and TAG contents in individual *Chlamydomonas reinhardtii* cells via SCRS. **a** Dynamics of starch, protein and TAG contents in the bulk biomass as measured by conventional approaches. **b** Temporal alteration of the averaged SCRS sampled from 60 cells over three culture replicates at each time points along the 16 time points. Reference Raman spectra of starch (corn starch), protein (bovine serum albumin V) and TAG (triacylglycerol 48:3) are shown above the SCRS for comparison. The contents of starch (**c**), protein (**d**) and TAG (**e**) of individual cells were derived using PLSR models, and the averaged contents in the population (*Y* axis; two of the three culture replicates were used for calibration and one for validation) was plotted versus the corresponding value measured with conventional methods at the population level (*X* axis). PLSR: partial least square regression. TLC–GC–MS: Thin-layer chromatography coupled with gas chromatography–mass spectrometry. *R* ^2^: correlation coefficient

To test whether the contents of starch, protein and TAG can be simultaneously quantified at the single-cell level via SCRS, the stress response process of *C. reinhardtii* under nitrogen depletion was employed as a model (Additional file 1: Figure S1a). To validate the new method, conventional approaches were separately used, via the aforementioned “one procedure per target compound” paradigm, to measure starch, protein and TAG contents based on metabolite mixture extracted from bulk microalgal biomass (“Methods”). The results revealed distinct temporal dynamics of these energy-storage compounds (Fig. 1a). Specifically, the starch content of the population increased by > 50-fold, starting from 6.04 ± 2.98 mg/g dry weight (DW) at 0 h to 323.58 ± 7.78 mg/g DW during Day 1, and then plateaued until a slight decrease to 284.79 ± 2.98 mg/g DW at Day 8. TAG content also increased (although to a much lower level than starch), from 1.20 mg/g DW at 0 h to the maximum of 35.0 mg/g DW at Day 6. However, the protein content sharply decreased from 565.90 ± 11.67 mg/g DW at 0 h to 225.86 ± 13.15 mg/g DW at Day 2, and then gradually reduced to 160 mg/g DW during the next 6 days (Fig. 1a).

The change in neutral lipid content was validated by Nile Red staining under confocal fluorescence microscopy, which revealed that the number of lipid bodies in cells increased along the process (Additional file 2: Figure S2). Transmission electron microscopy further revealed that the number and average size of starch granules significantly increased during the process, while lipid bodies gradually emerged and then merged into larger ones (Additional file 3: Figure S3). Moreover, lipid unsaturation degree, as assessed by mass unsaturation ration *N*_C=C_/*N*_CH2_ of the mixed fatty acids in the cellular extract via gas chromatography–mass spectrometry (GC–MS) analysis, underwent an increase from 1 to 8 days (Additional file 4: Figure S4a). Thus during the 8 days, carbon storage mode of the cells had switched from a protein-central one to a starch-central one where TAG also made a contribution (albeit much smaller than starch).

### Simultaneous quantification of starch, protein, TAG contents and lipid unsaturation degree at the single-cell level via SCRS

From the liquid suspension cultures that underlie the above analysis, SCRS from 60 randomly selected live cells were also collected (20 from each of three biological replicate cultures; Fig. 1b), at each of 16 time points over 8 days. Based on the reference chemical Raman spectra of pure starch, protein and TAG molecules, five Raman bands for starch, three for protein and five for TAG were proposed as the marker bands for quantification (Table 1). Their intensity, when averaged over all cells at a time point, exhibits positive correlation with the starch, protein and TAG contents measured via conventional approaches (correlation coefficient *R* ^2^ ranging from 0.6976 to 0.9300 for starch, from 0.7638 to 0.9105 for protein, and from 0.7721 to 0.8974 for TAG; Table 1). Moreover, the ratio of unsaturated-to-saturated carbon–carbon bonds, as measured by the relative intensity between the Raman bands of 1658 cm^−1^ (allyl C=C stretches proportional to the amount of unsaturated C=C bonds) and 1441 cm^−1^ (Alkyl C–H_2_ bend proportional to the amount of saturated C–C bonds), was used to model the lipid unsaturation degree of the cells [27, 29]. Consistently, *I*_1658_/*I*_1441_ exhibits positive correlation with lipid unsaturation degrees measured by GC–MS (*R* ^2^ = 0.9096) (Additional file 4: Figure S4b), which validated the ability of *I*_1658_/*I*_1441_ to model the unsaturation degree of total lipids.

**Table 1:** The 13 reference Raman bands that are highly correlated with starch, protein and TAG contents in *Chlamydomonas reinhardtii* CC124 during the process of nitrogen depletion

Component	Raman bands (cm^−1^)	*R* ^2^	Assignments
Starch	478	0.6976	C–C–C deformation
	865	0.9300	C–C–H and C–O–C deformations
	940	0.8895	C–O stretching; C–O–C and C–O–H deformation; α-helix C–C backbone
	1049	0.8308	C–O and C–C stretching; C–O–H deformation
	1127	0.8862	C–O and C–C stretching; C–O–H deformation
Protein	1003	0.7638	Phenylalanine ring breath; carotene C–H bending
	1586	0.9105	Phenylalanine
	1610	0.8850	C=O stretching of protein amide I; –NH_2_
Triacylglycerol^a^	972	0.8438	ν (C–C) wagging
	1265	0.8783	Alkyl=C–H
	1441	0.8974	Alkyl C–H_2_ bend
	1658	0.7721	Allyl C=C stretches
	2851	0.8902	C–H_2_, C–H_3_ asymmetric and symmetric stretches

Correlation coefficient (*R* ^2^) between averaged intensity of the Raman bands derived from SCRS and the corresponding quantitative trait shown

^a^Correlation coefficients calculated based on post-1d datasets, since the TAG content of *C. reinhardtii* cells during the first day of nitrogen depletion was very low

On the other hand, to take advantage of the rich and comprehensive information content in the SCRS, a chemometric multivariate method called partial least square regression (PLSR) was employed for predicting starch, protein and TAG contents of individual cells based on the full spectra of SCRS. PLSR is a method for developing multivariate calibration models for testing the correlations between the investigated properties and spectroscopic data. The normalized fingerprint region (393.8–1801.4 cm^−1^) and the hydrocarbon region (2701.6–3051.6 cm^−1^) were extracted for PLSR modeling due to their richness in information content. For each time point, two of the triplicate cultures were used as training dataset and the remaining one as test dataset for model validation. For example, for starch content, the PLSR model was established using the averaged SCRS of 20 cells in a biological replicate and the corresponding starch content measured by the amyloglucosidase/α-amylase method.

**Fig. 2:**
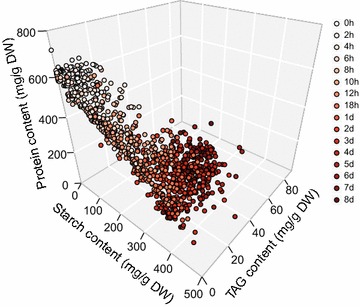
Starch, protein and TAG contents of individual *Chlamydomonas reinhardtii* cells along the process of nitrogen depletion. Each data point represents one cell, with color indicating the time point when the SCRS was acquired

The full spectrum-based PLSR model for starch content featured coefficient value (*R* ^2^) of 0.9966 for the calibration dataset and 0.9766 for the validation dataset, with the overall coefficient value (*R* ^2^) at 0.9892, suggesting the high accuracy in modeling starch content in single cells (Fig. 1c). Similarly, the PLSR models for protein and TAG contents in single cells were built and validated, achieving overall *R* ^2^ of 0.9924 and 0.9686, respectively (Fig. 1d, e). Each of the starch, protein and TAG contents derived via the full spectrum, when averaged from that of the individual cells as predicted by SCRS, was highly consistent with those experimentally determined from the bulk of microalgal biomass (Pearson correlation, *ρ* = 0.9984, 0.9988 and 0.9988, respectively; *P* < 0.01). Simultaneous visualization of the starch, protein and TAG contents of each of the 960 *C. reinhardtii* cells sampled over the 16 time points revealed the temporal landscape for microalgal energy storage compounds in the population at single-cell resolution (Fig. 2).

Collectively, these results underscore the advantages of full spectrum-based modeling of metabolite contents versus that based on the just a few marker Raman bands (Table 1), as it provides higher accuracy in measurement yet without replying on reference chemical spectra. Since they are generally applicable for *C. reinhardtii* CC124 cells, the datasets and scripts for building and validating the PLSR models can serve as valuable community resources (Additional file 5: Dataset S1, Additional file 6: Dataset S2, Additional file 7: Dataset S3, Additional file 8: Appendix S1). In conclusion, SCRS is able to simultaneously and accurately quantify starch, protein and TAG contents and lipid unsaturation degree at the single-cell level; moreover, the approach can be expanded to include other abundant metabolites in the cell.

### Heterogeneity of starch, protein, TAG contents and lipid unsaturation degree among individual cells

**Fig. 3:**
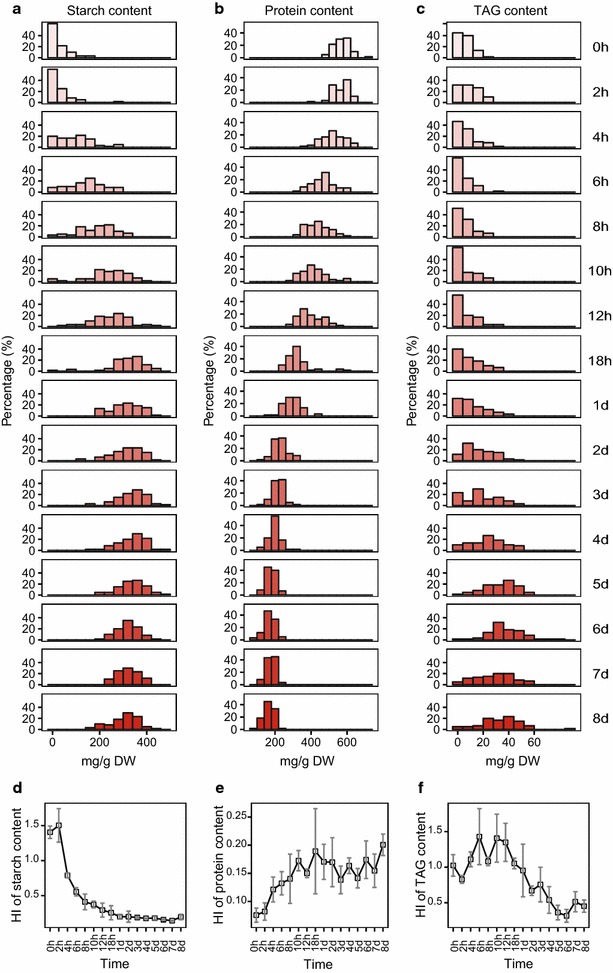
Phenotypic heterogeneity within the *Chlamydomonas reinhardtii* populations. **a**–**c** Distribution of single-cell starch, protein and TAG contents in the population at each of the 16 time points. *X* axis is the predicted starch, protein or TAG content in a cell (mg/g DW) and *Y* axis is the frequency (%) of such cells. *DW* dry weight. **d**–**f** Heterogeneity index (HI, i.e., relative standard deviation, RSD) of starch, protein or TAG contents in the populations at each of the 16 time points. Error bars represent the standard deviation from three biological replicates of culture at each time point

The ability to model the four phenotypes at the single-cell level allows measurement of their degree of among-cell heterogeneity in a given population. The heterogeneity index (HI) of a quantitative phenotype or trait is defined as the RSD (relative standard deviation) of individual cells within the population. For each of the four phenotypes, the phenotypic frequency distribution indicated a high degree of heterogeneity in energy-storage compounds, which was prevalent in the *C. reinhardtii* population regardless of its state (Fig. 3a–c; Additional file 4: Figure S4c).

However, the temporal change of HI was quite distinct among the four phenotypes. For starch, while the average content was increased, HI decreased sharply during Day 1 and then stayed at the lowest level for the remaining 7 days (Fig. 3d). For protein, while the average content decreased, HI increased during the first 18 h and then stabilized for the next 7 days and was always much lower than HIs of starch or TAG at each time point (Fig. 3e). For TAG, HI exhibited a high level of fluctuation during the first 12 h and then decreased afterward (Fig. 3f). As for lipid unsaturation degree, the HI stayed largely stable at a relatively low level along the full course of observation (Additional file 4: Figure S4d).

### Sampling depth affects the accuracy of metabolite content and HI measurements

**Fig. 4:**
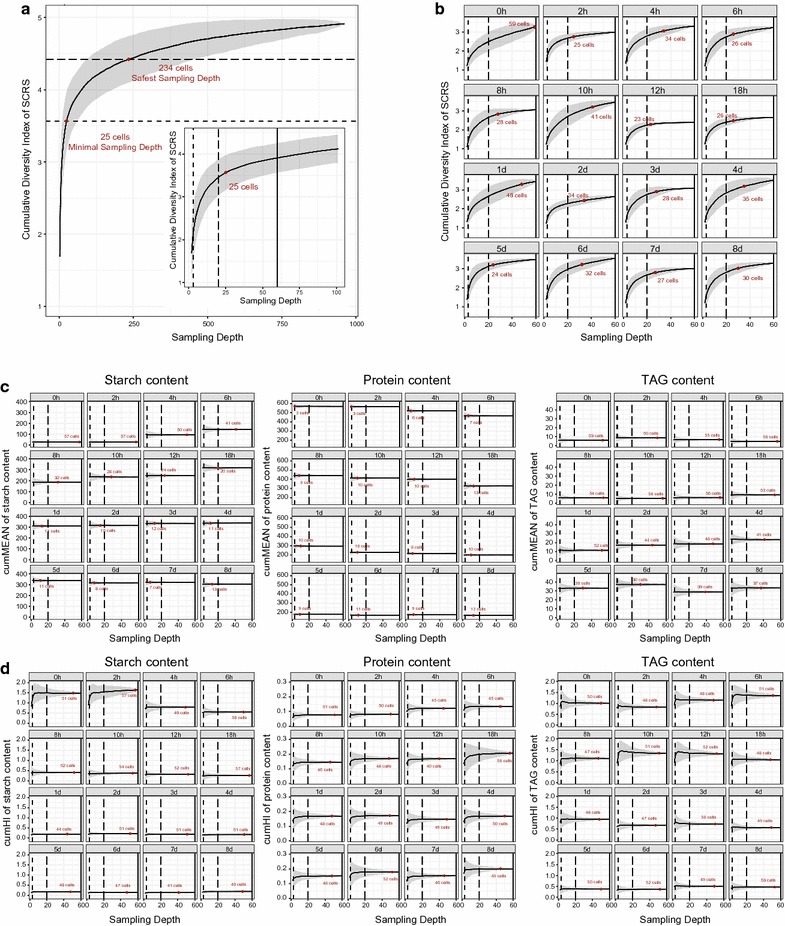
Effect of sampling depth on SCRS-based measurements. Quantitative relationship between sampling depth and the observed diversity of SCRS for an *in silico* population that includes all the cells sampled for SCRS over the 16 time points (**a**) or for the actual populations sampled for SCRS at each time point (**b**). At each sampling depth, 1000 permutations of sampling trials were performed, and the mean and standard deviations of the cumulative diversity index (DI) were calculated to estimate the extent to which the diversity of SCRS was observed and variation of the observation. In addition, quantitative relationship between sampling depth and the population average measurements of single-cell quantitative traits (**c**) and of their heterogeneity (**d**) is shown. Similarly, at each sampling depth, 1000 permutations of sampling trials were performed, and the cumMean and cumHI were calculated to estimate the observed values of quantitative traits and the variation of such observations. Gray areas represent the standard deviation of such measurements from the 1000 trials. The sampling depths of 3, 20 and 60 cells, each highlighted by a vertical line, were used as examples to quantitatively assess how the choice of sampling depth affects the accuracy and reliability of measuring the traits. For **a** and **b**, the minimal sampling depth, defined as the depth when no more than 1% of gain in cumulative DI will be gained by sampling one more cell (“Methods”; Additional file 9: Figure S5a, S5b), is highlighted for the 960-cell virtual population and for each time point. For **c** and **d**, the minimal sampling depth, defined based on the boundary condition of deviation of no more than 5% from the true means or true HI of metabolite contents (“Methods”; Additional file 9: Figure S5c, S5d), is highlighted for each time point. Minimal sampling depth was highlighted with red point and the corresponding text

Due to the inherent heterogeneity among cells in any population, phenotype measurements derived from SCRS might be potentially affected by the depth of sampling. Yet, the link between the accuracy of measurement and sampling depth remains untested. To quantitatively evaluate the observed diversity of SCRS at a particular sampling depth, a series of concepts were introduced. Briefly, (i) the Euclidian distance between any pair of SCRS sampled from a given population was used to measure the degree of divergence between the two SCRS; (ii) diversity index (DI), i.e., the maximal Euclidian distance among the set of SCRS sampled from a cellular population, was proposed to quantify the observed diversity of SCRS; (iii) cumulative DI is defined as the mean of the DI from a number of (*N* = 1000 here) simulated sampling trials. In the end, a saturation analysis plot was devised that depicts the relationship between cumulative DI and sampling depth, so as to provide a theoretical basis for rational assessment and selection of sampling depth (Fig. 4a, b).

First, we assembled a virtual pool of SCRS consisting of all the 960 SCRS collected over the 16 time points starting from the onset of nitrogen depletion, which presumably includes all possible SCRS for the *C. reinhardtii* strain under the given culture condition. The observations of DI over 1000 *in silico* trials of SCRS acquisition from this virtual pool of SCRS were compared over each sampling depth, which ranged from 2 to 960 (Fig. 4a). The results revealed that the higher the sample depth, the closer is the cumulative DI to the full SCRS diversity in the population (i.e., the more completeness in sampling the SCRS diversity); however at and after a certain threshold of sample depth, the cumulative DI would reach saturation where the SCRS diversity sampled no longer further increases. For this virtual dataset of 960 cells, the sampling depth required to reach 90% of the full SCRS diversity sampled is 234 cells, which is termed the “safest sampling depth” for a given strain under a particular culture condition. Moreover, the choice of sampling depth greatly affects the cumulative DI and thus the degree to which the acquired SCRS collection captures the full diversity of SCRS: for example, at the sample depth of 3, 20 and 60 cells, the cumulative DI is 2.12, 3.46 and 3.91, which correspond to 43, 70 and 80% of the theoretical maximal diversity of SCRS sampled (i.e., 4.91) (inset of Fig. 4a). On the other hand, in reality, one might never be able to thoroughly and exhaustively sample the SCRS space of a given isogenic cell population. Even for the 960-cell SCRS collection pooled from 16 time points here, the total SCRS diversity captured is still just an approximation of the “full” diversity. In fact, it is possible that no upper bounds are present for certain populations. Therefore, a definition of a “minimal sampling depth” that is not dependent on the total number of cells actually sampled is also valuable. To quantitatively determine whether the cumulative DI of an SCRS collection is saturated at a certain sampling depth, a term called “rate of cumulative DI” is defined, which measures the degree of cumulative DI increase with stepwise elevation of sampling depth (“Methods”). The parameter of “minimal sampling depth” is thus designated as the sampling depth at a cutoff of 1% for rate of cumulative DI, i.e., at this particular sampling depth, no more than 1% of increase in cumulative DI will be gained by sampling one more cell (“Methods”). The “minimal sampling depth” of the 960-cell collection is 25 (Additional file 9: Figure S5a), which is, not surprisingly, much lower than the “safest sampling depth” of 234.

The 960-cell virtual pool of SCRS consists of cells from 16 distinct time points; thus, it represents the theoretical upper limit of SCRS diversity for a strain under a particular condition. In practice, a cellular population sampled for SCRS at a particular time point may represent only a subset of the 960-cell SCRS pool. To test whether and how the relationship between the observed SCRS diversity and sampling depth changes over the various time points, the observations of DI over 1000 *in silico* trials of SCRS acquisition from each of the 16 time points were compared over each sampling depth (from 2 to 60; Fig. 4b). The results revealed that sampling depth can greatly affect cumulative DI. For example, at the sampling depth of 3, the observed cumulative DI ranges from 1.44 to 1.84, which are merely 45–62% (raised to 76–94% at the depth of 20 cells) of those at the depth of 60 cells. Thus, the sampling depth of 3 provides a highly incomplete and thus biased view of overall SCRS diversity at any of the time points, whereas in contrast the sampling depth of 20 or 60 cells yields a much better performance (Fig. 4b). Based on the definition above, the minimal sampling depth at each of the 16 time points was computed (Additional file 9: Figure S5b; also highlighted on Fig. 4b). They varied from 23 (at 12 h) to 59 (at 0 h), which suggested that the actual sampling depth of 60 in this experiment was sufficient at each of the time points.

For any quantitative trait at the single-cell level, assuming it follows normal distribution in an isogenic population, the mean and the degree of heterogeneity (or heterogeneity index; HI) are its most fundamental features. To provide a rational basis for determining the proper sampling depth of SCRS, we then tested the link between sampling depth and the measurements of mean and HI for the quantitative traits of interest. To test how the sampling depth influences the accuracy of measuring the metabolic contents and their respective HI for a given population state, we proposed the cumulative mean (cumMean) and cumulative HI (cumHI) as the observed mean and HI that were derived from 1000 *in silico* trials of SCRS acquisition at a particular sampling depth. At each of the trials, cells were randomly selected from the 60 cells at a certain sampling depth for SCRS acquisition, which were used to derive the mean and HI of quantitative traits (e.g., starch content, protein content and TAG content) as above. Their respective standard deviation errors over the 1000 trials (gray areas in Fig. 4c, d) were then computed to estimate the degree of variabilities in measurements of mean and HI.

For the measurement of population-averaged metabolite contents, at a low sampling depth, such as three cells per sample, for each of starch, protein and TAG under each of the 16 time points, experimental measurements of contents exhibit much higher variability than those at a higher sampling depth of 20 or 60. Thus, the measurement at a sampling depth of three cells would be usually of low reliability, poor accuracy and can sometimes deviate from the actual mean (Fig. 4c). However, with the increase of the sampling depth, the measurements rapidly converge toward the actual mean and the standard deviation errors gradually turn lower (gray areas in Fig. 4c), which indicates that measurements of starch, protein and TAG contents would be of much higher accuracy and excellent reliability under higher sampling depth such as 20 or 60 cells. In fact, the minimal sampling depth for each of the three phenotypes at each of the time points was calculated, based on the boundary condition of deviation of no more than 5% from the true means of metabolite contents (“Methods”; Additional file 9: Figure S5c). Clearly, the minimal sampling depth is not only time point dependent, but phenotype specific: for protein content, they vary from 3 (0, 2 h) to 13 (18 h, 8 days) (average of 9), while for starch content, they range from 7 to 57 (average of 25), with the time points before 18 h all higher than 20; in contrast, for TAG content, the values are generally even higher, ranging from 30 (5, 6 days) to 56 (6, 10, 12 h) (average of 47; Additional file 9: Figure S5c; also highlighted in Fig. 4c). Such variation originated from the distinct degrees of heterogeneity for the phenotypes.

For measurement of HI of the starch, protein and TAG contents, at a low sampling depth such as three cells per sample, the mean HI calculated from 1000 simulated experiments at most of the time points (in particular 0 and 2 h) significantly deviated from the actual HI, suggesting that HI measurements under low sampling depth would rarely be accurate at such a sampling depth (Fig. 4d). Moreover, under all the 16 time points, at a low sampling depth such as three cells per sample, the HI measurements show great variability, indicating the measurement results would be of low reliability and poor accuracy and likely deviating from the actual HI. However, with the increase of the sampling depth, the measurements rapidly converge toward the actual HI and the standard deviation errors gradually turn lower (gray areas in Fig. 4d), which indicates that measurement of the heterogeneity of metabolite contents is of much higher accuracy and excellent reliability under high sampling depth, such as 20 or 60 cells. Similar to the measurement of the mean content, the minimal sampling depth for each of the three HI at each of the time points was calculated (“Methods”; Additional file 9: Figure S5d). Interestingly, for each of starch, protein and TAG, the minimal sampling depth for HI are all within a narrow range of 40–57 (Additional file 9: Figure S5d; also highlighted in Fig. 4d). This suggests that to accurately profile the HI of a phenotype, generally higher minimal sampling depth is required.

In conclusion, the formal definition of sampling depth and the associated terms and saturation curves provide a theoretical basis for rational selection of proper sampling strategy for SCRS analysis. The simulation based on actual data proved that our strategy of sampling totally 60 SCRS (20 cells in each of triplicate cultures) at each time point provides an adequate coverage of the diversity of SCRS at the time point and thus yields accurate and reliable estimation of SCRS heterogeneity, as well as the content and HI measurements of starch, protein and TAG within a population.

### SCRS barcode for tracking the dynamics of the product profile

To facilitate strain screening or bioprocess monitoring via SCRS, a signature barcode derived from SCRS was proposed that consisted of two panels, one for population-averaged traits and the other from the degree of among-cell heterogeneity. The barcode is based on the 13 marker Raman bands that include 478, 865, 940, 1049 and 1127 cm^−1^ for starch, 1003, 1586 and 1610 cm^−1^ for protein, 972, 1265, 1441, 1658 and 2851 cm^−1^ for TAG. Moreover, *I*_1658_/*I*_1441_ was included to indicate lipid unsaturation degree (Table 1).

**Fig. 5:**
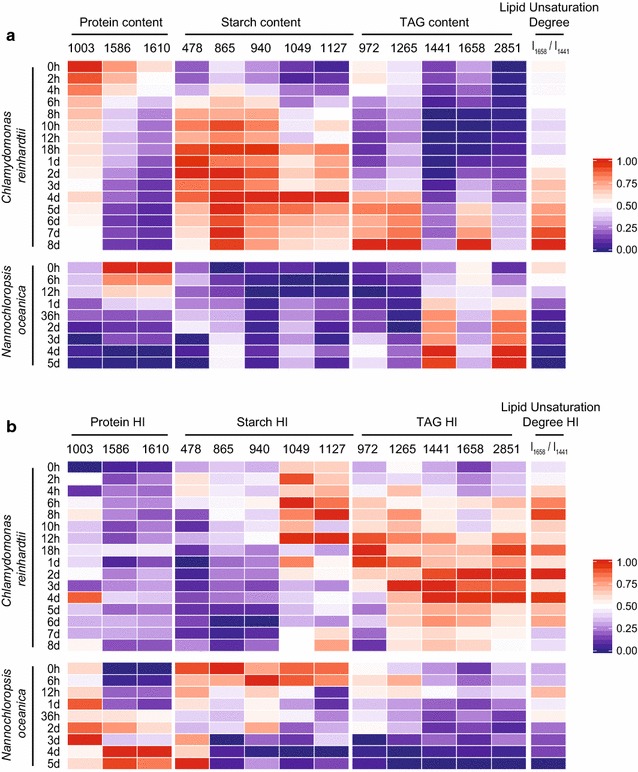
SCRS barcodes of *Chlamydomonas reinhardtii* CC124 and *Nannochloropsis oceanica* IMET1 under nitrogen depletion conditions. Population average (**a**) and heterogeneity index (**b**) of protein content, starch content, TAG content and lipid unsaturation degree (*I*_1658_/*I*_1441_) based on the 13 marker Raman bands in averaged SCRS of three biological replicates of culture at each time point are shown, which revealed graphically the distinct dynamic features of these key traits between the starch biosynthetic process of *C. reinhardtii* and the TAG-accumulating process of *N. oceanica* under nitrogen depletion conditions. The color of barcode indicates the relative intensity of Raman bands and was assigned based on the normalized intensity value within each Raman band

As proof of concept, the barcode was employed to unveil the dynamics of main energy-storage compounds and their among-cell heterogeneity in *C. reinhardtii* CC124 and an oleaginous industrial microalga *N. oceanica* (strain IMET1; Additional file 1: Figure S1b), both under the stress of nitrogen deprivation. Panel I defines the averaged population-level features (Fig. 5a). For example, for *C. reinhardtii* CC124 cells during nitrogen deprivation, the marker Raman bands for both starch and TAG exhibited an upward tendency in intensity, while those for protein exhibited an opposite trend, which were consistent with the population-level measurements. On the other hand, the SCRS barcode of *N. oceanica* IMET1 revealed that a large amount of TAG, yet little starch, was accumulated under nitrogen deprivation, which was consistent with previous reports [7, 23]. Furthermore, comparison of the *I*_1658_/*I*_1441_ section of SCRS barcode suggested that the lipids *C. reinhardtii* and *N. oceanica* produced under nitrogen deprivation differed in property: increase in relative abundance of unsaturated lipids in the former (as indicated by the gradual increase of *I*_1658_/*I*_1441_ ratio), yet rapid decrease (particularly during the first day) of unsaturated lipids in the latter (Fig. 5a). Such a temporal shift in the unsaturation degree of lipids synthesized upon the onset of nitrogen deprivation is supported by our published time series lipidomics data for *N. oceanica* IMET1 [30].

Panel II of the barcode defines the corresponding HI for each of the quantitative traits in Panel I (Fig. 5b). For example, for *C. reinhardtii* CC124 cells under nitrogen deprivation, HI of most starch-related Raman bands exhibited a slight upward tendency at the early phase and gradually decreased afterward, which was largely consistent with the findings derived from the full spectrum of SCRS (Fig. 3d). For HI of TAG-related Raman bands, an upward trend was observed at the early phase, which was followed by a downward trend after around 4 days. This suggested that for TAG content, the among-cell heterogeneity increased first and then decreased. As for *N. oceanica* IMET1 under nitrogen deprivation, HI of protein-related Raman bands showed an upward tendency, yet on the opposite that of TAG-related Raman bands (as well as *I*_1658_/*I*_1441_) exhibited a downward tendency, suggesting decrease in among-cell heterogeneity in TAG content and lipid unsaturation degree during the process. Thus by graphically highlighting the temporal changes of metabolite contents and their heterogeneity, comparison of the SCRS barcodes unveiled the shared and distinct biosynthetic features between microalgal species.

### Applicability of the method under a wide range of cell-storage conditions

In many circumstance, fresh living cells may be unavailable prior to SCRS acquisition. In fact, freeze drying (i.e., lyophilization), which works by freezing the biomass and then reducing the surrounding pressure to allow the frozen water to sublime directly from the solid phase to the gas phase, is the most common storage condition for algal cells. To test whether the method is robust under common cell-storage conditions beyond suspension culture, wet *C. reinhardtii* cell pastes preserved in a − 80 °C refrigerator and dry *C. reinhardtii* powders lyophilized by vacuum freezing dryer were, respectively, analyzed by the SCRS approach. The results from simultaneous quantification of starch, protein, TAG contents and lipid unsaturation degree were compared to those from suspension culture.

**Fig. 6:**
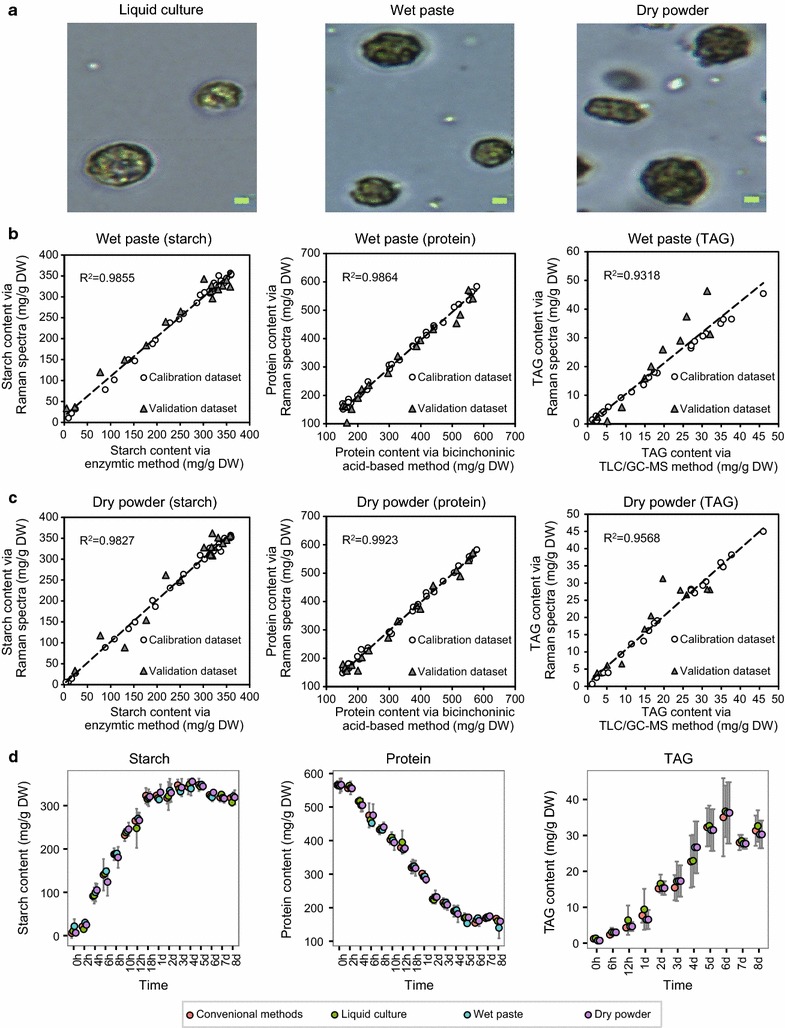
Quantification of starch, protein and TAG contents via single-cell Raman spectra from wet algal paste and dry algal powder. **a** Integrity of individual algal cells under the three algal cell-storage conditions of liquid suspension culture, wet algal paste and dry algal powder (Bar = 2 μm). The PLSR models for quantifying starch, protein and TAG contents via SCRS were built for cells in the form of wet algal paste (**b**) or dry algal powder (**c**). **d** Measurements of starch, protein and TAG contents for each of the three algal cell-storage conditions were highly consistent between the conventional method and the SCRS-based approach. PLSR: partial least square regression. TLC–GC–MS: thin-layer chromatography coupled with gas chromatography–mass spectrometry. *R* ^2^: correlation coefficient. Larger error bar in TAG measurement might be caused by the relative low level of TAG content in *C. reinhardtii* cells and the multiple largely manual operations (e.g., lipid extraction and thin-layer chromatography) in determining the TAG content of the cellular biomass

Briefly, a small amount of wet algal paste or dry algal powder was first suspended in ddH_2_O and then intact cells under the microscope were subject to acquisition of SCRS (Fig. 6a). The PLSR models to quantify starch, protein and TAG by Raman spectra of wet algal paste or dry algal powder were established and validated similarly to that of live cells in liquid suspension culture. For wet algal paste, SCRS-based quantification of starch, protein and TAG contents was accurate and reliable, as demonstrated by the high coefficient values (*R* ^2^) for both calibration datasets and validation datasets (the overall *R* ^2^ reached 0.9855, 0.9864 and 0.9318; Fig. 6b; Additional file 10: Table S1). The performance was similar for dry algal powders, since the overall *R* ^2^ reached 0.9827, 0.9923 and 0.9568 respectively (Fig. 6c; Additional file 10: Table S1). Importantly, no significant difference was found in starch, protein and TAG contents among those measured by conventional methods and those predicted via SCRS in liquid suspension culture, wet paste or dry powder (Student *t* test, *P* > 0.1; Fig. 6d).

As for lipid unsaturation degree, the *I*_*1658*_/*I*_1441_ derived from SCRS as measured via both wet algal paste and dry algal power showed significant positive correlation with that measured by GC–MS (*R* ^2^ = 0.6342 and 0.6233 respectively). Thus, SCRS acquired from wet algal paste and dry algal power can also be interpreted for lipid unsaturation degree of individual cells. Therefore, the SCRS approach appears to be generally applicable to a wide range of cell-storage conditions, including those most commonly used in preservation and storage of biological samples (e.g., cryopreservation or lyophilization).

### Conclusion and discussion

"Ramanome", i.e., a collection of SCRS randomly sampled from a cellular population at a given condition and time, captures the “metabolomic state” of cells in a rapid, label-free, non-invasive and single-cell resolution manner [18]. Here, we demonstrated that the SCRS of *C. reinhardtii* is able to simultaneously quantify four key phenotypes of microalgae-based production: starch content, protein content, TAG content and lipid unsaturation degree. The marker peaks that can model the quantity of these major intracellular energy-storage compounds were identified and their performance was critically assessed (Table 1). Quantitative models based on the full spectrum of SCRS provide higher accuracy than those based on the marker peaks; thus, such models can be established and then shared as a valuable community resource for SCRS-based analysis and screening of microbial cell factories and biological processes mediated by them.

Phenotypic heterogeneity among cells, a universal trait of cellular populations, can dramatically affect cellular processes and thus lead to profound biological implications or practical applications. For example, substantial cell-to-cell variations might be advantageous to the robustness of the cellular population and subject to evolutionary selection [31, 32]. In contrast, for many industrial biotechnological processes, a high degree of heterogeneity can cause low yields, suboptimal productivity or even failures [33, 34]. In this study, the degree of heterogeneity of energy-storage compounds (i.e., starch and TAG) decreased sharply at the early phase and stabilized at low level later under the stress of nitrogen deprivation. The observation reflects the dynamic response and eventual homeostasis of *C. reinhardtii* cells under stress and might serve as a direct indicator for bioprocess monitoring and control. Therefore, the ability to track the heterogeneity of multiple physiological parameters (i.e., starch content, protein content, TAG content and lipid unsaturation degree) in a rapid, label-free and simultaneous manner should be of value to the dissection of mechanisms underlying cellular systems’ performance, and to the optimization and control of bioprocesses.

Actually, single-cell Raman spectra can potentially capture two levels of heterogeneity for an isogenic cellular population: intracellular heterogeneity and intercellular heterogeneity. On one hand, when the diameter of the laser spot is smaller than the cell size, one SCRS theoretically samples only a particular region of a cell. This leads to SCRS variation among regions in a cell, i.e., the intracellular heterogeneity. On the other hand, at any given state of an isogenic population, phenotypes among cells can vary greatly, due to difference in cell cycle phases, micro-environmental conditions and even spontaneous mutations [33]. This leads to intercellular heterogeneity.

To assess the intracellular heterogeneity, a recent study compared three Raman spectra acquisition modes, i.e., single spectra, spectra of images and integrated Raman spectra, for the classification of large eukaryotic cells (i.e., T lymphocyte and pancreatic cell lines). To overcome the effects of intracellular heterogeneity, acquisition of integrated Raman spectra that covered an 8 × 8 µm^2^ region of a cell was suggested for classification of cells [35]. A related study estimated a minimum number of local spectra sampling within a cell (i.e., “sampling density” instead of “sampling depth”) for characterizing single cells of lymphocytes (~ 20 μm of cell diameter), and claimed that 30 measurements of Raman spectra from random locations within a cell showed a performance that is similar to that of acquiring a whole-cell Raman image [36]. Both of these studies stressed that intracellular heterogeneity affected the ability of Raman spectra to characterize or classify cells and proposed ways to overcome these intracellular heterogeneities. However, they did not address intercellular heterogeneity within a cellular population. In contrast, our manuscript mainly addressed intercellular heterogeneity, i.e., the effect of sample size (i.e., “sampling depth”) per cellular population on the performance of SCRS in characterizing cells, using microalgal cells (of ~ 10 μm in diameter and thus are much smaller than lymphocytes) as a model.

Due to the presence of intracellular heterogeneity, large cells typically require acquisition of multiple SCRS to properly characterize them (e.g., the lymphocyte cells mentioned above). However, for small size cells, the overall functional state can be described by a single SCRS, especially measured under aqueous conditions. In this study, SCRS of *C. reinhardtii* cells were acquired via Raman tweezers which used the 532 nm laser and 50 × objective to create an optical trap for holding individual cells under aqueous condition, and the same laser was used to acquire the whole-cell spectra. Importantly, when the cell was held in the single-beam gradient force trap, the cell under the aqueous condition was rolling in random orientation. Therefore, our measurements represent the averaged spectra of a whole cell instead of a specific region in the cell.

The presence and importance of intercellular heterogeneity, both for diversity of SCRS as a whole and for a particular SCRS-derived phenotype, demands methods to quantitatively determine minimal sampling depth. Here, we showed that sampling depth, a parameter generally ignored in past studies, greatly influences the accuracy and reliability of SCRS-based measurement. Moreover, we proposed two kinds of “minimal sampling depth” for a given population “state”: (i) for exploring diversity of SCRS, the minimal sampling depth is defined as the depth when no more than 1% of increasement in cumulative DI is gained by sampling one more cell; (ii) for measuring the mean and HI of a particular phenotype such as metabolite content, minimal sampling depth is defined based on the boundary condition of deviation of no more than 5% from the true means or true HI. The methodology introduced here to rationally determine minimal sampling depth for a given population is generally applicable. It should not only guide the design of future studies, but also enable retrospective assessment of past studies that either sampled at only a few cells per population [24–26] or offered no rationale for their choice of sampling depth for a particular system [19, 22, 23, 27, 28]. Our study also suggests that efforts to automate SCRS acquisition from a given sample will be highly valuable, as it might allow routine analysis at sampling depth of dozens or even hundreds of cells.

Due to the rich information harbored in the SCRS, the type and number of phenotypes that can be tackled by SCRS are theoretically unlimited. For example, as many valuable compounds carry characteristic Raman signal, this method should be able to rapidly provide a landscape-like view of biosynthetic capability of cells at single-cell resolution. Moreover, SCRS can be interpreted for a much greater range of phenotypes beyond metabolite profile: for example, SCRS has recently been used to characterize substrate metabolism [37, 38], metabolic activity [39], stress response [18, 40] and interspecies interactions [37, 41]. Therefore to gauge and fulfill the potential of the SCRS approach, one research direction is to probe whether, and to what degree, the plethora of phenotypes can be simultaneously measured or modeled via SCRS. On the other hand, considering that acquisition of SCRS is non-destructive and cells of targeted SCRS can be subsequently isolated by Raman-activated cell sorting (RACS) [42–44], novel applications such as Raman-activated mutant screening, which links SCRS-based phenotyping to sequencing at single-cell resolution, might be possible and deserve investigation.

Finally, as it requires only a trace number of cells, yet its performance is insensitive to variation in sample storage conditions, SCRS approach might find particular value in circumstances where cells grow slowly (or are yet to be cultured), SCRS acquisition cannot be performed onsite, or only cryopreserved or lyophilized biomass is available. This good feature can be taken advantage of to greatly expand the application of SCRS and Raman-activated cell sorting and sequencing technologies [45].

## Methods

### Strains and growth conditions

*Chlamydomonas reinhardtii* strain CC124 was grown in standard Tris acetate phosphate (TAP) medium at 25 °C under continuous lighting (approximate 150 µmol photons m^−2^ s^−1^) and bubbled with air to ensure mixing and prevent settling. Cells grown to late log phase were re-inoculated to a final concentration of 2 × 10^6^ cells mL^−1^ in nitrogen-depleted TAP medium, in which NH_4_Cl was omitted. The re-inoculation was carried out in triplicate cultures. One milliliter of cell culture was collected at 0 h (right after inoculation), 2, 4, 6, 8, 10, 12, 18 h and 1, 2, 3, 4, 5, 6, 7 and 8 days for analysis. *Nannochloropsis oceanica* IMET1 were grown in a modified f/2 liquid medium with 4 mM NO_3_^−^ under continuous light (approximately 50 μmol photons m^−2^ s^−1^) at 25 °C and induced in nitrogen depletion f/2 medium, in which NO_3_^−^ was omitted. Cultures in triplicate at 0, 6, 12 h, 1 day, 36 h and 2, 3, 4 and 5 days were sampled.

### Quantification of starch, protein, TAG content and lipid unsaturation degree in cultures

The algal biomass was lyophilized via vacuum freezing dryer. The starch content of the dry algal biomass was quantified using an enzymatic starch assay kit (Megazyme K-TSTA 07/11). Briefly, samples of about 20 mg dry algal power were treated with 80% ethanol to remove sugars, hydrolyzed into soluble maltodextrins with thermostable α-amylase and then digested into d-glucose with amyloglucosidase. The generated glucose was exposed to a reagent containing glucose oxidase, peroxidase and 4-aminoantipyrine and then its content quantified spectrophotometrically at a wavelength of 510 nm.

The total protein content of algal cultures was measured as previously reported [7]. Briefly, approximately 10 mg of lyophilized algal biomass was hydrolyzed in 200 μL lysis buffer (1 M sodium hydroxide, NaOH) and then incubated at 80 °C for 10 min in a water bath. Then, 800 μL ddH_2_O was added to the hydrolysate to bring the volume to 1 mL. Cellular debris was centrifuged at 12,000*g* for 30 min before the supernatant was transferred to a new tube. The extraction was repeated two more times and all the supernatant extracts were pooled together. Then, the total protein in the supernatant was determined by the BCA Protein Assay kit (cat no. cw0014s).

Thin-layer chromatography coupled with gas chromatography–mass spectrometry (TLC–GC–MS) and gas chromatography–mass spectrometry (GC–MS) was performed to analyze the TAG content and the lipid unsaturation degree of the total lipids. The procedures mainly included total lipid extraction, TLC, transesterification and GC–MS analysis [7]. Specifically, total lipids of about 30 mg lyophilized algal powder were extracted with 6 mL chloroform:methanol (2:1, v/v) and recovered in chloroform:methanol (2:1, v/v). For TAG quantification, around 0.3 mg lipid extract was loaded onto 10 × 20 cm silica TLC plates (Merck KGaA, Darmstadt, Germany). TAG was separated, visualized and scraped from the plate. Then TAG was extracted with chloroform:methanol (2:1, v/v) from TLC powder and used to prepare fatty acid methyl esters (FAMEs) as previously described [7]. Briefly, 20 μL 2 mg mL^−1^ methyl tridecanoate (C13Me), 200 μL chloroform:methanol (2:1, v/v) and 300 μL 5% (v/v) HCl:methanol were added to TAG extracts, which were transesterified in tightly sealed vials at 85 °C for 1 h. Finally, the FAMEs extracted were analyzed on an Agilent 7890-5975C gas chromatography mass spectrometer fitted with an HP-INNOWAX 30 m × 0.25 mm × 0.25 μm column. The FAMEs were quantified using pentadecane as the internal standard and C8–C24 FAMEs mixture as FAMEs standards. The TAG content was determined by conversion from the content of FAMEs.

To measure the lipid unsaturation degree of total lipids from bulk microalgal biomass, 1 mg total lipid extract was directly transesterified to FAMEs, which were then analyzed via GC–MS as described above. The abundance of individual fatty acids in the total lipid extract was calculated based on the GC–MS data. The lipid unsaturation degree was calculated via the ratio between the number of C=C bonds and the number of CH_2_ bonds of total fatty acids as previously described [23, 27, 29]. Briefly, the average mass unsaturation ration *N*_C=C_/*N*_CH2_ can be represented as ∑(niN_C=C_^*i*^)/∑(niN_CH2_^*i*^), where ni refers to the relative abundance of the individual fatty acids in the total lipid extract (mg/mg dry weight), *N*_C=*C*_^*i*^ refers to the number of C=C bonds of the individual fatty acids and *N*_C=C_^*i*^ refers to the number of CH_2_ bonds.

### Fluorescent microscopy and transmission electron microscopy

For microalgal cells sampled at 0, 12 h and 1, 3, 5 and 7 days, lipid droplets were first stained by Nile Red (Sigma, USA). Briefly, 5 μL of stock Nile Red solution (50 μg/mL) was added to 0.5 mL microalgae culture and incubated in the dark for 10 min. Fluorescence images were then acquired using an Olympus BX51 florescence microscope with a 100×/1.3 oil immersion objective and U-MWB2 mirror unit. For transmission electron microscopy, microalgae cells were sampled at 0 h and 3 and 5 days and then centrifuged (500*g*, 5 min). Cell pellets were fixed by 2.5% glutaraldehyde for 12 h at 4 °C and then washed three times with phosphate-buffered saline (PBS, pH7.4). After post-fixation in 1% osmic acid for 1 h, samples were again washed three times with PBS. The samples were dehydrated through an acetone series (30–95%; followed by pure acetone three times) and then embedded in Spurr’s resin. Micrographs of ultrathin sections were captured using a Hitachi H-7650 transmission electron microscope (Hitachi High-Technologies Co., Japan).

### Acquisition of single-cell Raman spectra

Single-cell Raman spectra were measured using a modified Horiba LabRam HR with an excitation wavelength of 532 nm [44]. For acquisition of SCRS from live cells, 1 mL of microalgal culture was collected at each of the time points. After centrifugation, cells were washed with ddH_2_O for three times and loaded into a capillary tube (50 mm length × 1 mm width × 0.1 mm height, Camlab, UK). The Raman spectra of 20 cells and four background sites in each of the three biological replicate cultures (i.e., 60 cells per time point) were randomly recorded. Briefly, an individual cell was trapped, photobleached and measured by a 532 nm laser with about 25 mW output power. Each Raman spectrum was acquired between 393.8 and 3341.3 cm^−1^ and the acquisition time was 2 s.

For preparation of wet algal pastes, 10 mL of each algal culture was centrifuged at 3000*g* and cell pellets preserved at − 80 °C until use. For preparation of dry algal powders, the wet algal pastes were lyophilized in a vacuum freezing dryer for 24 h and then stored at − 80 °C until use. Before SCRS acquisition, aliquots of the wet algal paste thawed at 4 °C or about 1 mg of dry algal powders were resuspended in 0.2 mL ddH_2_O by gentle shaking, and then SCRS of individual algal cells were acquired as described above.

### Analysis of single-cell Raman spectra

Raw SCRS were pre-processed for background subtraction, baseline correction and normalization with Labspec5 software (HORIBA JobinYvon Ltd., UK). Two information-rich regions of SCRS, the biochemical fingerprint region (393.8–1801.4 cm^−1^) and the hydrocarbon region (2701.6–3051.6 cm^−1^), were separately extracted for further analysis [46]. For both regions, a spectrum was normalized via division by its area. PLSR, a method for developing multivariate calibration models to study correlation between the investigated properties and spectroscopic data, was employed to predict the starch, protein and TAG contents from SCRS at both the population level and the single-cell level as previously described [23, 47]. At certain time points, the predicted starch, protein or TAG contents of a small number of cells were lower than the detection threshold (i.e., < 0 mg/g DW); these contents were regarded as 0 mg/g DW in subsequent analysis. the ratio of the intensity of the two Raman bands at 1658 cm^−1^ (cis C=C stretching mode which is proportionate to the amount of unsaturated C=C bonds) and 1441 cm^−1^ (CH_2_ scissoring mode which is proportionate to the amount of saturated C–C bonds) was employed to estimate the lipid unsaturation degree [23, 27, 29]. Procedures to quantify the starch, protein and TAG content via SCRS of wet algal paste and dry algal powder were established and validated in a manner similar to that of live microalgal cells in liquid suspension culture. PLSR models were produced using Matlab R2010a (Mathworks, USA).

### Quantifying the influence of sampling depth on measurement accuracy

The degree of divergence between the two SCRS was quantified via the Euclidian distance between any pair of SCRS sampled from a given population. Diversity index (DI), i.e., the maximal Euclidian distance among the set of SCRS sampled in an SCRS population, was proposed to quantify the observed diversity of SCRS for a particular state of a given cell population. Cumulative diversity index was proposed to estimate the observed SCRS diversity in a series of SCRS collections sampled at a particular depth for a particular state of the population. To perform saturation analysis, whether for a time point (60 cells) or for the collective cells from all the 16 time points (960 cells), an SCRS collection was acquired by randomly sampling the population for SCRS at a particular sampling depth that ranged from two to all cells, and such trials were performed for 1000 times at each sampling depth. At each sampling depth, the mean and standard deviation of the cumulative DI were calculated to estimate the extent to which the diversity of SCRS was observed and the variation of the observation. The mean and standard deviations were then, respectively, plotted against the sampling depth, so as to quantitatively model their interaction. To quantitatively determine whether the cumulative DI (cumDI) of an SCRS pool at each time point was saturated at a certain sampling depth, we defined the “rate of cumDI”:

$${\text{Rate of cumDI }}\left( N \right) \, = \frac{{{\text{cumDI}}_{N} - {\text{cumDI}}_{N - 1} }}{{{\text{cumDI}}_{N - 1} }}{ ,}$$

where *N*: sample depth; cumDI_N_: cumDI at the sampling depth of *N* cells for a population; cumDI_*N*−1_: cumDI at the sampling depth of (*N* − 1) cells for a population. For each of the time points, the relationship between the rate of cumDI and sampling depth was thus plotted. We set a cutoff of 1% for the rate of cumDI to define the “minimal sampling depth”, which means, at this particular sampling depth, no more than 1% of gain in cumDI will be gained by sampling one more cell.

To assess the influence of sampling depth on the measurement of traits and their respective among-cell heterogeneity, cumulative mean (cumMean) was proposed as the average of the quantitative traits (e.g., contents of starch, protein or TAG) modeled via SCRS based on a number of individual cells, and cumulative heterogeneity index (cumHI) as the degree of heterogeneity for each of the quantitative traits among the cells sampled, in a series of SCRS collections sampled at a particular depth for a particular state of the population. To perform saturation analysis at each time point, an SCRS collection was acquired (and mean and HI of the quantitative traits calculated) by randomly sampling the population for SCRS at a particular sampling depth that ranged from one to all cells, and such trials were performed 1000 times at each sampling depth. At each sampling depth, the cumMean and cumHI were calculated to estimate the observed values of quantitative traits and the variation of such observations. The cumMean and cumHI were then, respectively, plotted against the sampling depth, so as to quantitatively assess how the choice of sampling depth affects the accuracy and reliability of measuring the traits. To quantitatively assess the effect of sampling depth on the cumMean and cumHI, the relationship between standard deviation (from 1000 trials) and the sampling depth per population was plotted for each time point. The minimal sampling depth for computing mean contents and mean HI (of starch, protein or TAG) was determined based on a threshold of 5% in the standard deviation of the 1000 permutations, which corresponds to a deviation of no more than 5% from the true means of metabolite contents or their HI in each measurement.

## Additional files

**Additional file 1: Figure S1.** Growth curves of *Chlamdomonas reinhardtii* CC124 and *Nannochloropsis oceanica* IMET1 under the condition of nitrogen depletion (N–). The microalgal growth was tracked via OD_750_.

**Additional file 2: Figure S2.** Detection of neutral lipid accumulation in *Chlamdomonas reinhardtii* cells by Nile Red staining.

**Additional file 3: Figure S3.** Transmission electron microscopy images tracking the accumulation of lipid bodies and starch granules in *Chlamdomonas reinhardtii* cells under the condition of nitrogen depletion.

**Additional file 4: Figure S4.** Temporal dynamics of lipid unsaturation degree at the population level and the single-cell level.

**Additional file 5: Dataset S1.** Dataset for building and validating the PLSR model for starch content quantification in *Chlamydomonas reinhardtii* CC124 cells.

**Additional file 6: Dataset S2.** Dataset for building and validating the PLSR model for protein content quantification in *Chlamydomonas reinhardtii* CC124 cells.

**Additional file 7: Dataset S3.** Dataset for building and validating the PLSR model for TAG content quantification in *Chlamydomonas reinhardtii* CC124 cells.

**Additional file 8: Appendix S1.** Matlab script for building and validating the PLSR models for quantification of starch, protein and TAG contents.

**Additional file 9: Figure S5.** Computation of “Minimal Sampling Depth”.

**Additional file 10: Table S1.** Performance of PLSR models for starch, protein and TAG quantification under the cell-storage conditions of liquid-suspension culture, wet paste and dry powder.

## Abbreviations

cumHI: cumulative heterogeneity index
cumMean: cumulative mean
DW: dry weight
DI: diversity index
FAMEs: fatty acid methyl esters
GC–MS: gas chromatography–mass spectrometry
HI: heterogeneity index
MS: mass spectrometry
PLSR: partial least square regression
R^2^: coefficient value
RSD: relative standard deviation
SCRS: single-cell Raman spectra
TAG: triacylglycerol
TAP: tris acetate phosphate
TLC–GC–MS: thin-layer chromatography coupled with gas chromatography–mass spectrometry
