# Supplementary figures and images for: Label-free, simultaneous quantification of starch, protein and triacylglycerol in single microalgal cells
Source: Biotechnol Biofuels. 2017 Nov 17;10:275. doi: 10.1186/s13068-017-0967-x (PMC5693592; doi:10.1186/s13068-017-0967-x)

Figure S1

(a)

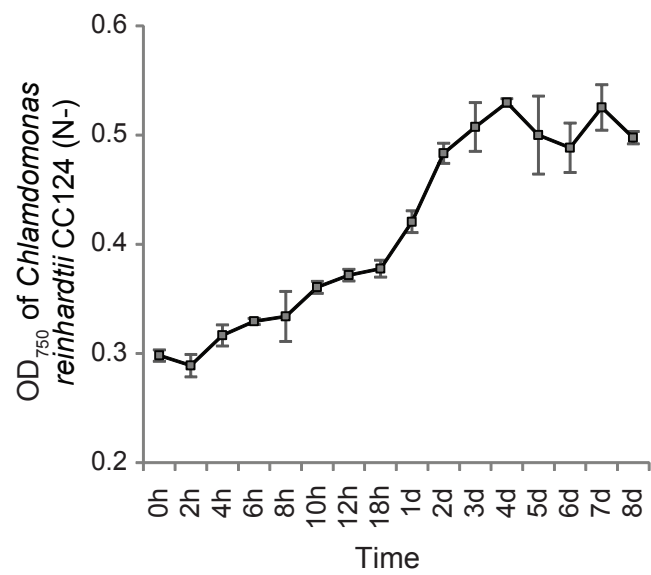

(b)

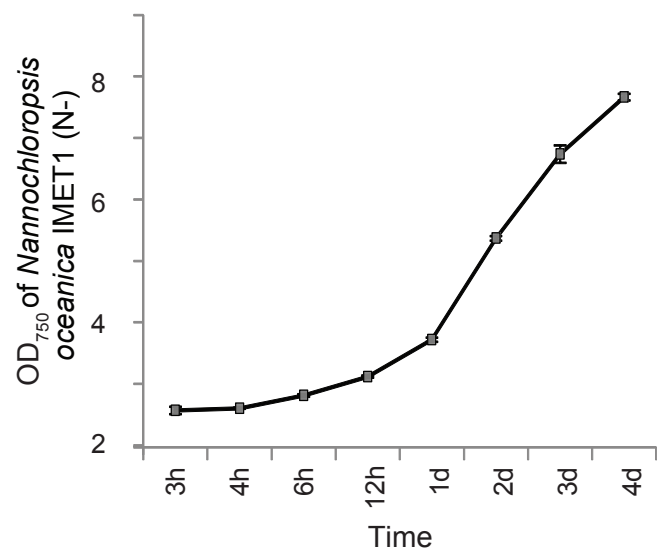

Supplement: Supplementary file 1 — Additional file 1: Figure S1. Growth curves of Chlamdomonas reinhardtii CC124 and Nannochloropsis oceanica IMET1 under the condition of nitrogen depletion (N–). The microalgal growth was tracked via OD750. [file 13068_2017_967_MOESM1_ESM.pdf]

Figure S2

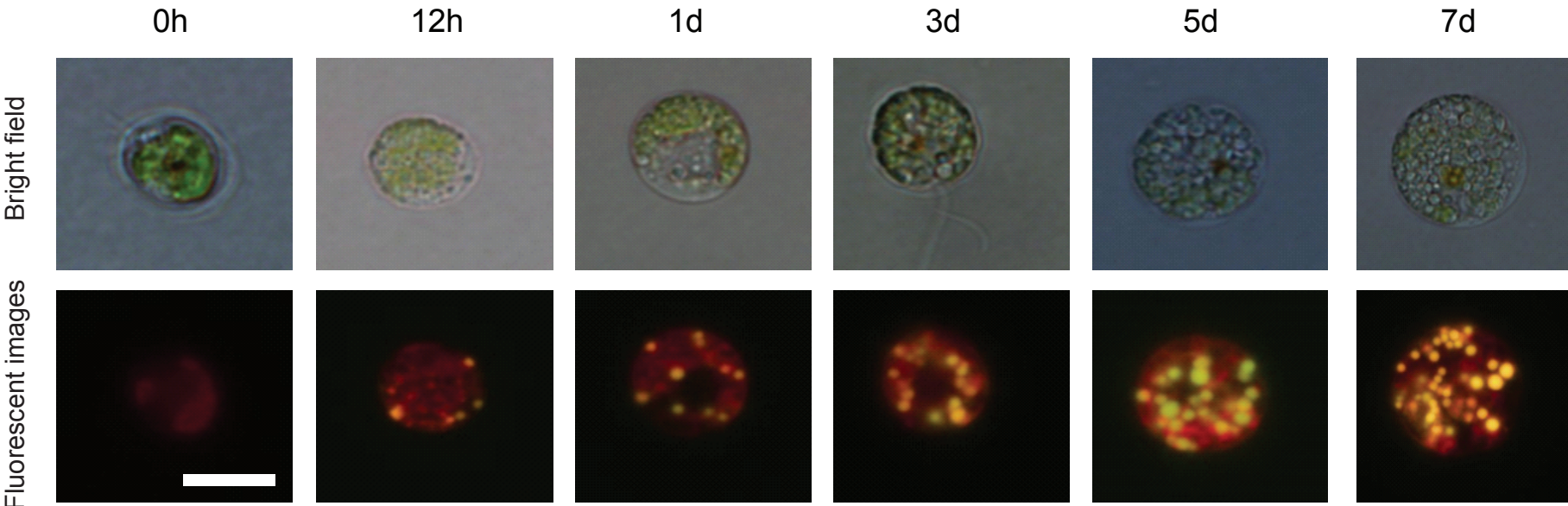

Supplement: Supplementary file 2 — Additional file 2: Figure S2. Detection of neutral lipid accumulation in Chlamdomonas reinhardtii cells by Nile Red staining. [file 13068_2017_967_MOESM2_ESM.pdf]

Figure S3

(a)

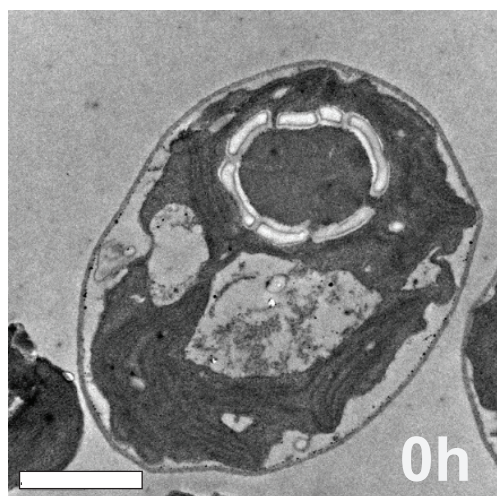

(b)

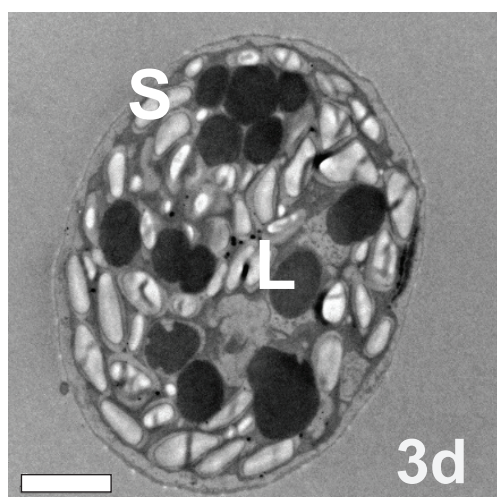

(c)

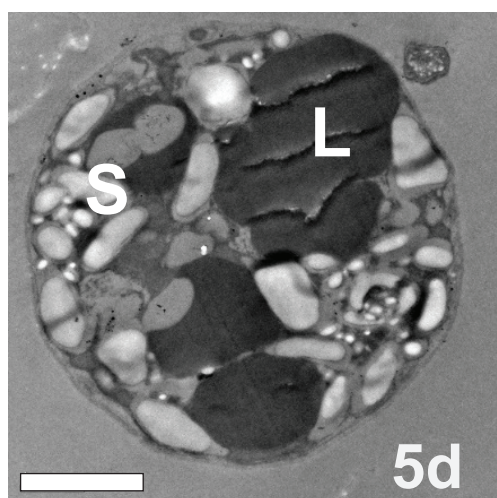

Supplement: Supplementary file 3 — Additional file 3: Figure S3. Transmission electron microscopy images tracking the accumulation of lipid bodies and starch granules in Chlamdomonas reinhardtii cells under the condition of nitrogen depletion. [file 13068_2017_967_MOESM3_ESM.pdf]

Figure S4

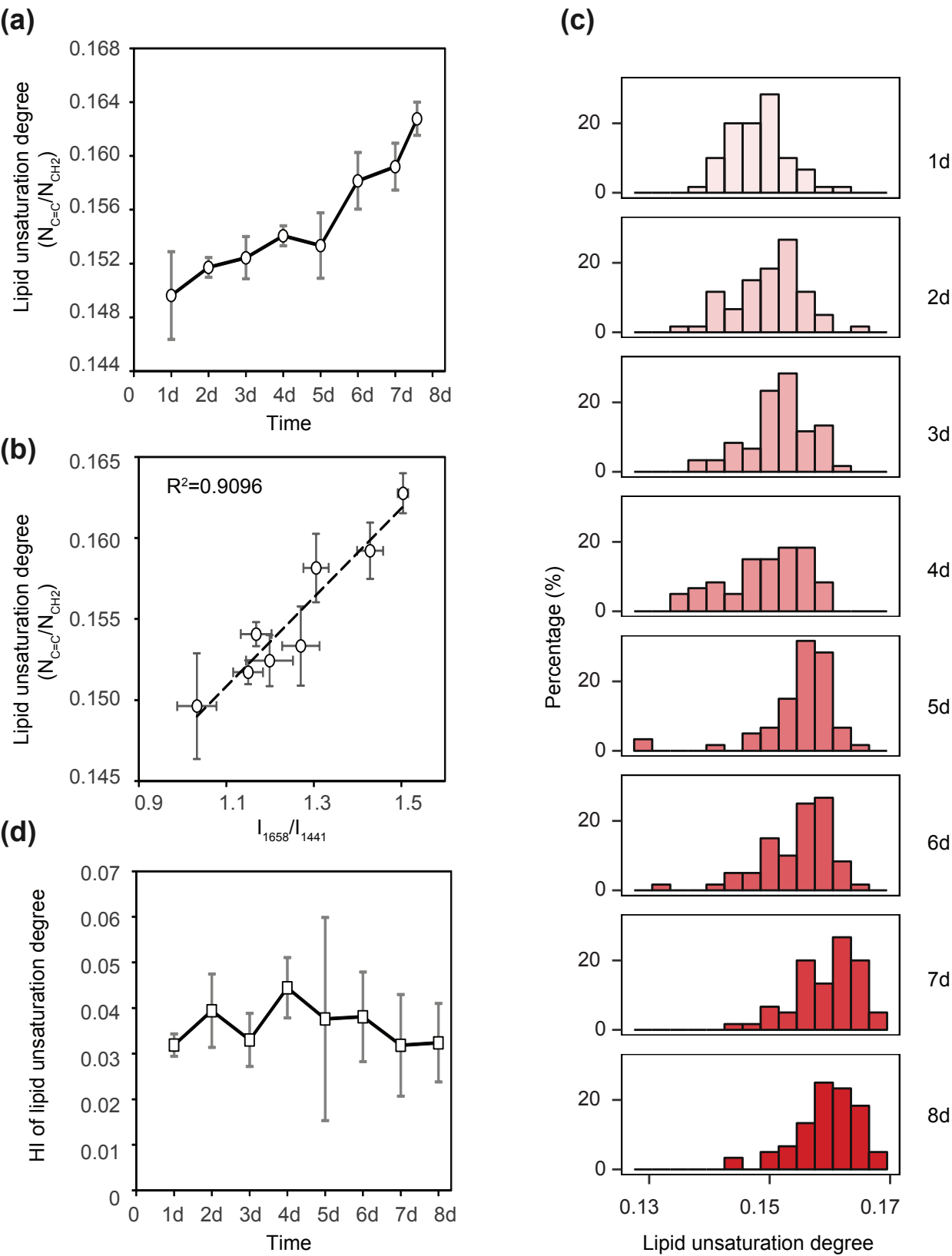

Supplement: Supplementary file 4 — Additional file 4: Figure S4. Temporal dynamics of lipid unsaturation degree at the population level and the single-cell level. [file 13068_2017_967_MOESM4_ESM.pdf]

Figure S5

(a)

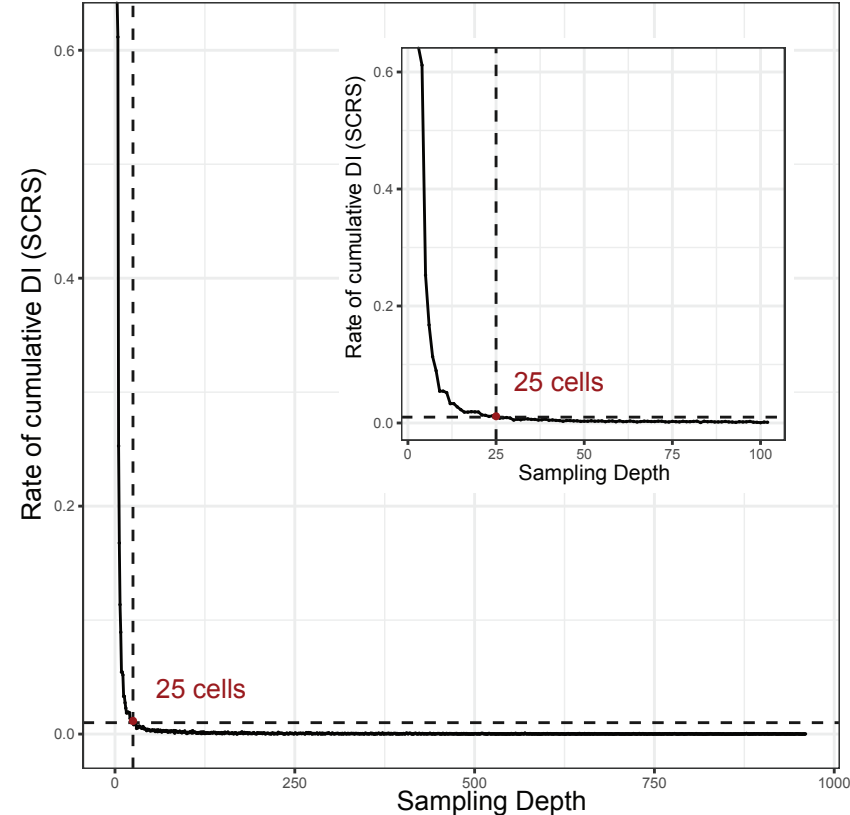

(b)

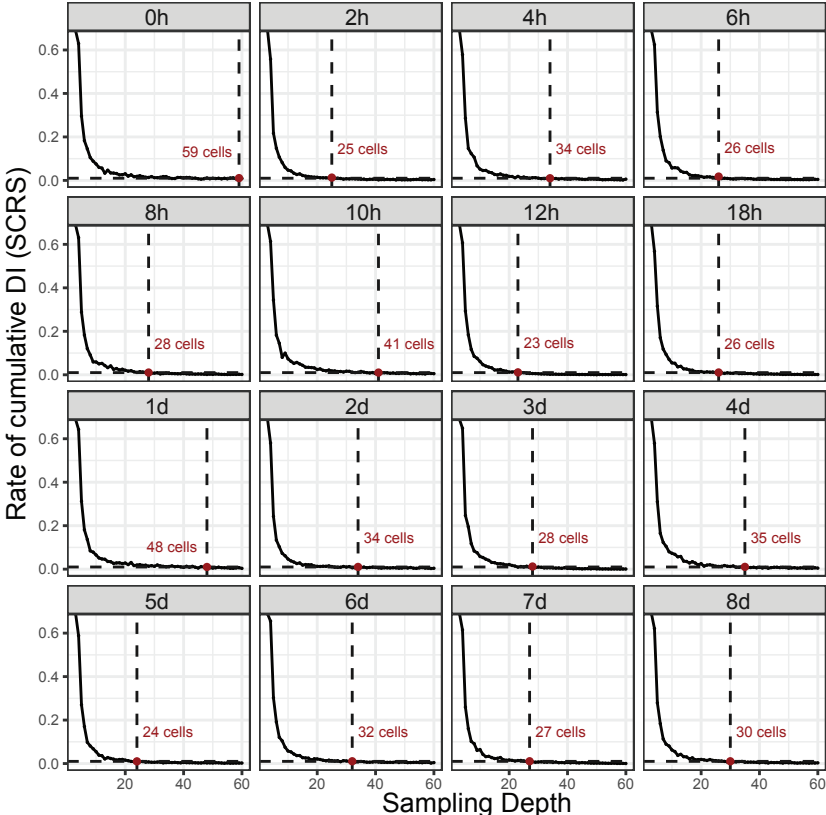

Figure S5

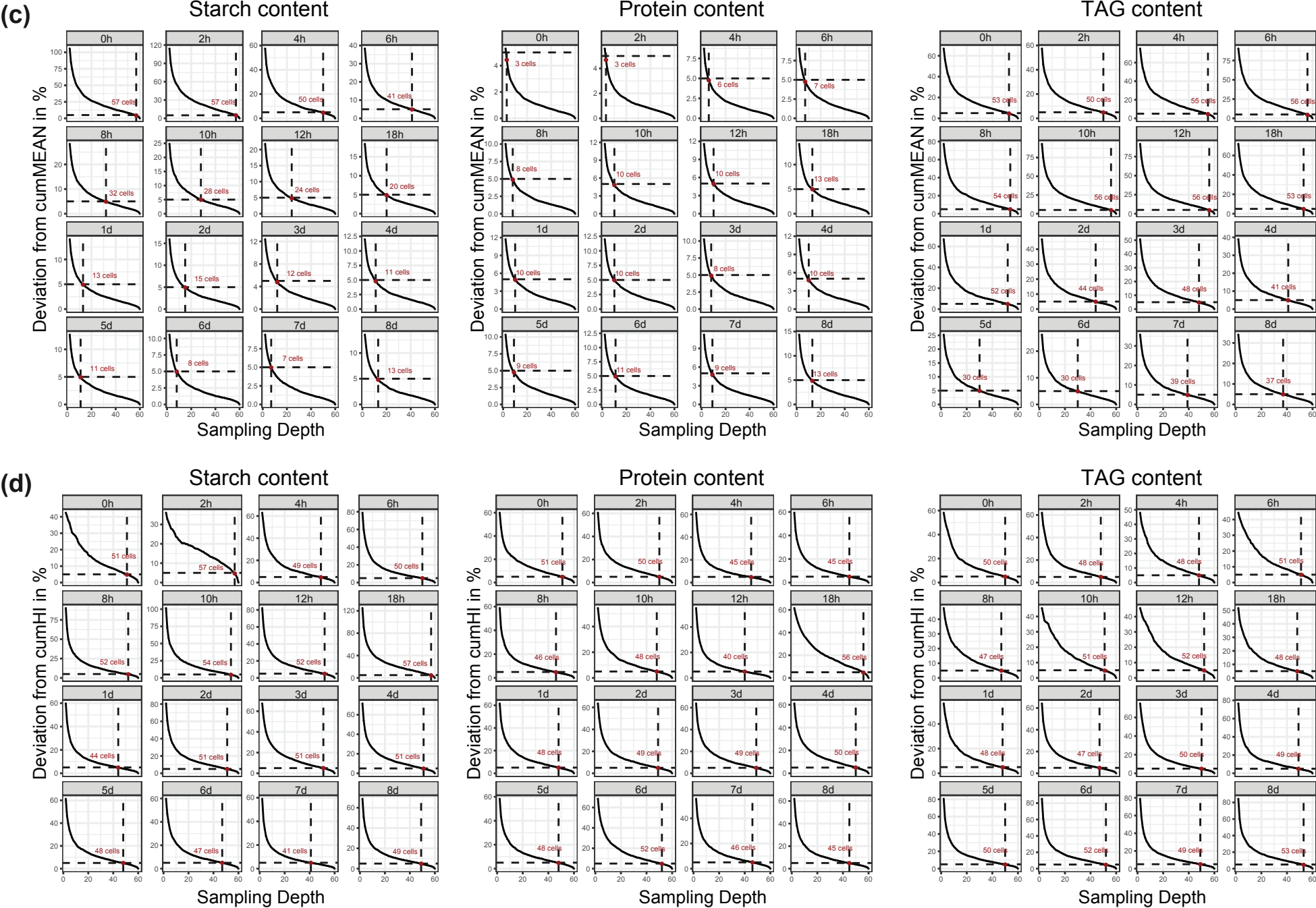

Supplement: Supplementary file 9 — Additional file 9: Figure S5. Computation of “Minimal Sampling Depth”. [file 13068_2017_967_MOESM9_ESM.pdf]
