# Supplementary material for: Label-free, simultaneous quantification of starch, protein and triacylglycerol in single microalgal cells
Source: Biotechnol Biofuels. 2017 Nov 17;10:275. doi: 10.1186/s13068-017-0967-x (PMC5693592; doi:10.1186/s13068-017-0967-x)
Supplement: Supplementary file 10 — Additional file 10: Table S1. Performance of PLSR models for starch, protein and TAG quantification under the cell-storage conditions of liquid-suspension culture, wet paste and dry powder. [file 13068_2017_967_MOESM10_ESM.docx]

**Table S1.** **Performance of PLSR models for starch, protein and TAG quantification under the cell storage conditions of liquid-suspension culture, wet paste and dry powder.** PCs: principal components; PCTVAR: Percentage of variations explained by these PCs; SCRS: Single-Cell Raman Spectra; R^2^: Correlation coefficient.

| PLSR models | | Starch | | | Protein | | | TAG | | |
| --- | --- | --- | --- | --- | --- | --- | --- | --- | --- | --- |
|  |  | Live cells | Wet paste | Dry powder | Live cells | Wet paste | Dry powder | Live cells | Wet paste | Dry powder |
| PCs | | 6 | 6 | 6 | 6 | 6 | 6 | 7 | 7 | 7 |
| PCTVAR(%) | SCRS | 95.75 | 91.55 | 92.21 | 95.48 | 92.00 | 92.62 | 97.46 | 95.26 | 95.95 |
|  | Starch /TAG/Protein | 99.66 | 99.61 | 99.65 | 99.85 | 99.57 | 99.68 | 99.82 | 99.78 | 99.62 |
| R^2^ | Calibration dataset | 0.9966 | 0.9965 | 0.9965 | 0.9985 | 0.9957 | 0.9968 | 0.9982 | 0.9978 | 0.9962 |
|  | Validation dataset | 0.9766 | 0.9757 | 0.9505 | 0.9813 | 0.9736 | 0.9852 | 0.9393 | 0.9094 | 0.8268 |
|  | Overall dataset | 0.9892 | 0.9855 | 0.9827 | 0.9924 | 0.9864 | 0.9923 | 0.9686 | 0.9318 | 0.9568 |
